# Review of the Safety and Clinical Considerations of Vasoconstrictor Agents in Dental Anesthesia During Pregnancy

**DOI:** 10.3390/jcm14134773

**Published:** 2025-07-06

**Authors:** Andrei Urîtu, Victor Bogdan Buciu, Ciprian Roi, Doina Chioran, Denis Mihai Serban, Nicolae Nicoleta, Elena Lavinia Rusu, Mihai Ionac, Mircea Riviș, Sebastian Ciurescu

**Affiliations:** 1 Doctoral School, “Victor Babes” University of Medicine and Pharmacy, E. Murgu Square, No. 2, 300041 Timisoara, Romania; andrei.uritu@umft.ro (A.U.); sebastian.ciurescu@umft.ro (S.C.); 2 Department of Anesthesiology and Oral Surgery, Research Center of Dento-Alveolar Surgery, Anesthesia and Sedation in Dental Medicine, “Victor Babes” University of Medicine and Pharmacy, Eftimie Murgu Sq. No. 2, 300041 Timisoara, Romania; ciprian.roi@umft.ro (C.R.); rivis.mircea@umft.ro (M.R.); 3 Departament of Anesthesiology and Oral Surgery, Research Center in Dental Medicine Using Conventional and Alternative Technologies, “Victor Babes” University of Medicine and Pharmacy, Eftimie Murgu Sq. No. 2, 300041 Timisoara, Romania; doina.chioran@umft.ro; 4 Department of Obstetrics-Gynaecology, Discipline of Obstetrics-Gynecology, “Victor Babes” University of Medicine and Pharmacy, E. Murgu Square, No. 2, 300041 Timisoara, Romania; denis.serban@umft.ro (D.M.S.); nicolae.nicoleta@umft.ro (N.N.); 5 Department of Microsurgery, Vascular Surgery and Scientific Research Methodology, “Victor Babes” University of Medicine and Pharmacy, E. Murgu Square, No. 2, 300041 Timisoara, Romania; mihai.ionac@gmail.com

**Keywords:** epinephrine, levonordefrin, felypressin, dental anesthesia, pregnancy, vasoconstrictors, fetal

## Abstract

**Background**: The use of vasoconstrictors in dental anesthesia during pregnancy raises clinical concerns due to their potential effects on uteroplacental blood flow and fetal well-being. Despite widespread use, the safety profiles of agents such as epinephrine, levonordefrin, and felypressin remain insufficiently reviewed, particularly in isolation from local anesthetics. **Methods**: A systematic literature search was conducted using PubMed (MEDLINE) for studies published between January 2000 and May 2025, following PRISMA 2020 guidelines. Included studies assessed the use, pharmacokinetics, or outcomes of vasoconstrictor agents used in dental procedures during pregnancy. Articles were selected based on predefined inclusion criteria and synthesized narratively. **Results**: Out of 95 screened records, only six narrative reviews and three clinical guidelines met eligibility criteria. Epinephrine was the most frequently described agent, generally regarded as safe at low dental doses (1:100,000–1:200,000) when properly administered. Levonordefrin showed insufficient safety data and was associated with potential uterine vasoconstriction. Felypressin was contraindicated due to its oxytocic properties and high risk of inducing uterine contractions. **Conclusions**: Among vasoconstrictors, epinephrine remains the only agent with an acceptable safety profile in pregnancy when used correctly. Levonordefrin lacks adequate evidence, and felypressin poses clear risks. Until further clinical trials are available, individualized risk assessment and adherence to obstetric and dental guidelines are critical for ensuring maternal and fetal safety.

## 1. Introduction

Maintaining oral health during pregnancy is essential not only for maternal well-being but also for reducing the risk of adverse pregnancy outcomes. Periodontal disease and dental infections, if left untreated, can contribute to systemic inflammation and have been associated with preterm birth and low birth weight [1,2]. Accordingly, dental procedures requiring local anesthesia should not be postponed solely due to pregnancy. However, the pharmacological safety of substances used in dental practice (particularly vasoconstrictor agents, adjuvants to anaesthesia) remains a topic of clinical concern.

Vasoconstrictors, such as epinephrine, levonordefrin, and felypressin, are frequently combined with local anesthetics to prolong their duration of action, reduce bleeding, and enhance depth of anesthesia [3,4]. These agents act primarily through alpha-adrenergic or vasopressinergic pathways, inducing localized vasoconstriction at the site of injection [3,4]. However, their systemic absorption, although typically minimal, can have significant physiological implications during pregnancy, a state characterized by increased cardiac output, decreased vascular resistance, and altered hormonal regulation of vascular tone [5,6].

Epinephrine, the most commonly used vasoconstrictor in dentistry, is classified as Food and Drug Administration (FDA) Pregnancy Category C, indicating that risk to the fetus cannot be ruled out. While low-dose epinephrine in local anesthesia is generally considered safe during pregnancy, concerns persist regarding potential uterine vasoconstriction, reduced placental perfusion, and exacerbation of hypertensive disorders such as preeclampsia [7,8]. Other vasoconstrictors, such as levonordefrin, a synthetic catecholamine, and felypressin, a vasopressin analog with known oxytocic effects, have limited safety data in pregnant patients [7].

Despite the widespread use of these agents, there is no consolidated synthesis of clinical data and professional guidelines specifically addressing the risks and safety profiles of vasoconstrictors used in dental anesthesia during pregnancy. Most reviews focus on the anesthetic component (e.g., lidocaine, articaine), with vasoconstrictors discussed only as adjuncts.

The purpose of this review is to explore and synthesize current evidence regarding the maternal and fetal effects and safety of vasoconstrictor agents used in dental anesthesia during pregnancy. It seeks to provide a comprehensive evaluation of the pharmacologic properties of these agents, including their mechanisms of action, potential for placental transfer, and associated clinical outcomes. Ultimately, the review aims to deliver evidence-based guidance to support informed clinical decision-making when managing pregnant patients who require dental procedures.

## 2. Materials and Methods

### 2.1. General Considerations

This systematic review was conducted in accordance with the PRISMA 2020 reporting standards, with the goal of evaluating the safety and clinical implications of vasoconstrictor agents used in dental anesthesia during pregnancy. The review focused exclusively on agents such as epinephrine, levonordefrin, and felypressin, excluding studies that addressed only the anesthetic compounds themselves (e.g., lidocaine, articaine) without evaluating the vasoconstrictor component specifically. This systematic review has not been registered.

### 2.2. Study Selection

A structured search of the PubMed (MEDLINE) database was performed to identify relevant studies published between 1 January 2000 and 10 May 2025. This timeframe was selected due to little innovation during the last few decades and the continuous utilization of already well-established techniques and medications—thus facilitating the gathering of a satisfactory number of studies. The search was executed using a combination of Medical Subject Headings (MeSH) and free-text terms related to vasoconstrictors, dental anesthesia, and pregnancy. The full search syntax included the following: (“epinephrine” OR “levonordefrin” OR “felypressin” OR “vasoconstrictor agents” OR “vasopressin analogs”) AND (“dental anesthesia” OR “dental procedures”) AND (“pregnancy” OR “pregnant women”).

No restrictions were imposed regarding study design; however, specific filters were applied to enhance the relevance and quality of the included literature. Only studies conducted on human subjects, published in English, and appearing in ISI-indexed journals were considered eligible, ensuring a foundation of peer-reviewed scientific rigor for this review.

In addition to the primary database search, relevant clinical guidelines from professional bodies—including the American Dental Association (ADA) and the American College of Obstetricians and Gynecologists (ACOG)—were manually searched and reviewed. References of selected articles were also screened to identify any additional eligible studies not captured through the database search.

Publications were deemed eligible for inclusion if they addressed the use of one or more vasoconstrictor agents during dental procedures in pregnant patients and provided information on maternal or fetal outcomes, pharmacokinetic or pharmacodynamic characteristics, or overall safety profiles. Conversely, findings were excluded if they focused exclusively on local anesthetics without evaluating the vasoconstrictor component, involved solely animal or in vitro research, or were not published in English or in ISI-indexed journals.

All retrieved records were imported into a structured review database. Titles and abstracts were screened independently by two reviewers (A.U. and V.B.). Full texts of potentially eligible studies were reviewed in detail, and relevant data were extracted, including: publication year, study type, type of patient population, vasoconstrictor(s) used, dosage and delivery route, trimester of administration, and any reported adverse maternal or fetal outcomes, including hypertension, preterm labor, or fetal compromise.

### 2.3. Study Design

Given the limited number of randomized controlled trials and the diversity of designs and outcomes, we have decided to declare our work as a narrative synthesis rather than a systematic one. The findings were grouped according to the type of vasoconstrictor, with comparative discussion based on clinical pharmacology, reported safety profiles, and expert consensus.

## 3. Results

The initial search of the PubMed (MEDLINE) database yielded a total of 95 records (published between 1 January 2000 and 10 May 2025) related to the use of vasoconstrictor agents in dental anesthesia during pregnancy. After removal of 23 duplicates and 2 inappropriately titled papers, 70 unique records were screened based on titles and abstracts. Of these, 32 were excluded for not meeting the predefined inclusion criteria, based on title and abstract. From the remaining 38 titles, 8 were excluded due to language non-compliance, 17 were excluded due to further inclusion and exclusion criteria not being met, and 7 for noncompliance to methodology that would make them appropriate for inclusion in our study, such as in vitro or animal studies. After database evaluation, 6 narrative reviews were included.

Additionally, 2 professional clinical guidelines from the ADA and ACOG were included to contextualize clinical recommendations, after excluding similar duplicate papers (n = 3). However, the limited number of eligible papers (n = 8) underscores a significant gap in direct clinical evidence on vasoconstrictor safety during pregnancy. Moreover, the evidence base is heavily weighted toward epinephrine, with minimal data available for levonordefrin and felypressin.

**Figure 1:**
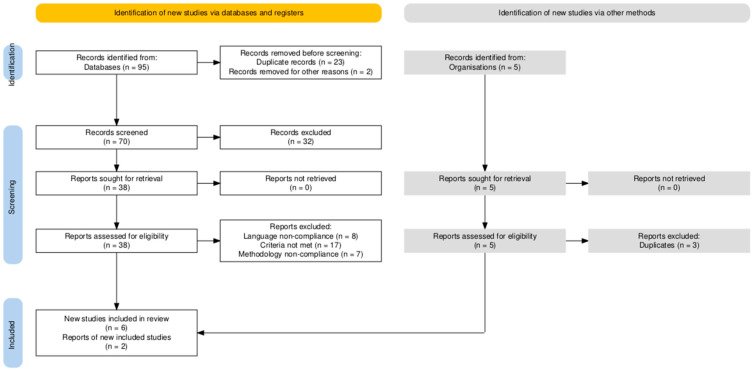
PRISMA Flowchart of Study Selection. PRISMA: Preferred Reporting Items for Systematic Reviews and Meta-Analyses. The diagram details the stages of identification, screening, and inclusion of studies sourced from both databases and supplementary manual searches from professional organizations. Reasons for exclusion at each stage, such as duplicate records, language non-compliance, unmet inclusion criteria, or methodological inadequacies, are explicitly provided to ensure transparency and reproducibility of the review process. (Diagram adapted from the PRISMA 2020 statement).

The systematic search and selection process of studies included in this review, depicted according to the PRISMA 2020 guidelines, is illustrated in Figure 1.

The literature directly addressing the safety of vasoconstrictor agents in dental anesthesia during pregnancy remains limited, with most existing reviews focusing primarily on the anesthetic component. To synthesize the most relevant findings, this review identified six key papers and two authoritative clinical guidelines that explicitly evaluated the maternal and fetal implications of epinephrine, levonordefrin, and felypressin use in pregnant patients. Table 1 provides a detailed overview of these sources, highlighting the type of evidence, population characteristics, the trimester of exposure where available, the vasoconstrictors assessed, and the principal conclusions drawn regarding safety and clinical applicability.

The use of vasoconstrictor agents in dental anesthesia during pregnancy has long raised questions among both clinicians and patients, largely due to concerns about uteroplacental perfusion and potential fetal compromise. However, a growing body of evidence [9,10,11,12,13,14,15] suggests that these concerns may be unfounded when vasoconstrictors are used appropriately and in low concentrations. A well-regarded narrative review [7] concluded that epinephrine, when administered at typical dental concentrations (1:100,000 to 1:200,000), does not pose significant obstetric risk. The authors emphasized that the agent’s rapid metabolism (primarily via catechol-O-methyltransferase and monoamine oxidase in maternal tissues) substantially limits its placental transfer. As long as aspiration is properly performed to avoid intravascular injection, systemic absorption remains minimal, rendering the risk of uterine vasoconstriction negligible [7].

This reassuring conclusion is echoed in a more recent review from 2024 [9], which further supports the continued use of lidocaine with epinephrine as a standard anesthetic approach in pregnant dental patients. This paper reinforces not only the relative safety of this combination but also the need for caution with alternative vasoconstrictors, particularly those lacking robust reproductive safety data. They advocate for a risk-aware but pragmatic approach to dental care during pregnancy, noting that poorly controlled oral infections (if left untreated due to fear of anesthesia) may pose a far greater danger to maternal–fetal health than the agents used to manage them [9].

In contrast, other vasoconstrictors such as levonordefrin warrant a more cautious stance. Fayans et al. (2010), in a comprehensive review of dental pharmacology in pregnant and postpartum patients, advised against the use of levonordefrin during gestation. Their concerns stem from the compound’s potent α-adrenergic activity and the absence of adequate safety data in pregnant populations [10]. Levonordefrin’s potential to induce systemic vasoconstriction and elevate maternal blood pressure raises the theoretical risk of reduced uterine blood flow—particularly problematic in pregnancies already complicated by hypertensive disorders [10,11,12,13,14,15,16]. This pharmacologic profile sets levonordefrin apart from epinephrine, which, although also a sympathomimetic, has a more favorable safety profile and better-studied clinical track record [10,11,12,13,14,15,16].

Clinical perspectives align with these pharmacologic findings. Another manuscript [11] offered practical insights drawn from dental care of pregnant patients, emphasizing that epinephrine remains a viable and safe vasoconstrictor when used with careful technique. They pointed to the importance of dosage control (maximum recommended dose: 0.04 mg) [11]. This corresponds to approximately 4 mL of a 1:100,000 solution or 8 mL of a 1:200,000 solution. Please note that local anesthetic cartridge volume varies by country. In the United States, a standard dental cartridge contains 1.7 mL. Their recommendations were based on clinical experience rather than statistical trials, but they reflect a widely accepted consensus within dental practice that prudent use of epinephrine is both efficacious and safe across all trimesters [11,12,13,14,15,16,17].

When turning to less commonly used agents, felypressin emerges as a particularly problematic choice. Manautou and Mayberry (2023), in a rigorous evidence-based review, warned against its use in pregnancy due to its structural similarity to vasopressin and its known oxytocic properties [12]. Felypressin can stimulate uterine contractions, a pharmacodynamic effect that significantly limits its safety during mid to late gestation. While it is sometimes promoted for use in patients with cardiovascular disease due to a milder pressor effect, this advantage becomes irrelevant in the context of pregnancy, where uterine quiescence is paramount [18]. Another review reaffirmed the safety of epinephrine in standard dental concentrations, contrasting it directly with felypressin and reinforcing current clinical preferences [12].

Expanding the scope, a review [13] examined adverse effects following dental anesthesia across a broad population, including pregnant patients. The authors noted that systemic reactions to vasoconstrictors (especially in physiologically altered states such as pregnancy) require heightened awareness. They discussed levonordefrin in particular as a vasoconstrictor with more pronounced cardiovascular effects, most notably elevated blood pressure with reflex bradycardia due to its strong α1-adrenergic activity, cautioning that the lack of gestational safety data should warrant its avoidance unless no alternative is available. These findings suggest that not all vasoconstrictors are created equal and that pregnancy-specific pharmacovigilance remains essential [13].

Professional guidelines mirror these conclusions. The ADA (2023) clearly supports the use of epinephrine-containing anesthetics during pregnancy, provided that proper technique is used. These guidelines advocate for aspiration prior to injection, avoidance of overdose, and recognition of maternal physiologic changes that may affect drug metabolism and distribution. Importantly, they highlight that withholding treatment out of misplaced caution can result in more serious maternal or fetal complications due to untreated oral pathology [14].

This message is reinforced by the ACOG, which in Committee Opinion No. 569 states that dental procedures requiring local anesthesia can be safely performed during pregnancy—ideally in the second trimester, but not contraindicated in the first or third. ACOG specifically addresses epinephrine-containing anesthetics, noting that these are acceptable when clinically indicated and when appropriate safeguards are observed [15].

Taken together, the reviewed evidence and expert guidance evaluated and included in this study suggest that epinephrine, when used in standard dental concentrations and with adequate technique, is a safe and effective vasoconstrictor in pregnant patients. In contrast, both levonordefrin and felypressin present pharmacological profiles that raise sufficient concern to recommend their avoidance. These findings underscore the importance of individualized care, interdisciplinary collaboration, and adherence to established guidelines when planning dental interventions during pregnancy.

To aid clinical decision-making, a structured comparison of the three primary vasoconstrictor agents—epinephrine, levonordefrin, and felypressin—commonly encountered in dental anesthesia is presented in Table 2. This summary highlights critical pharmacological and clinical characteristics, including their mechanisms of action, systemic absorption profiles, placental transfer potential, cardiovascular effects, and recommendations from major clinical guidelines. Emphasis is placed on factors particularly relevant during pregnancy, such as uteroplacental risk and fetal safety, to support safe and evidence-based practice in dental care for pregnant patients.

As previously stated, epinephrine demonstrates the most favorable safety profile when used at standard dental concentrations (1:100,000–1:200,000), supported by rapid metabolism, low systemic absorption, and endorsement from both the ADA and ACOG guidelines [7,9,10,11,12,13,14,15]. In contrast, levonordefrin exhibits a more potent α-adrenergic vasoconstrictive effect, coupled with insufficient human safety data and limited guideline support, warranting cautious or restricted use [10,11,12,13]. Felypressin, a vasopressin receptor agonist with known oxytocic effects, poses a clear risk of uterine stimulation and is contraindicated during pregnancy [12,14,15]. This comparative analysis reinforces the preferential use of epinephrine in pregnant patients requiring dental anesthesia, while highlighting the clinical limitations and potential hazards associated with alternative agents.

## 4. Discussions

### 4.1. Physiological and Genetic Factors Affecting Vasoconstrictor Response in Pregnancy

Pregnancy induces a cascade of physiological adaptations that significantly alter the pharmacokinetics and pharmacodynamics of administered agents, including vasoconstrictors. Cardiovascular changes are particularly pronounced, with increases in blood volume and cardiac output of up to 50%, reductions in systemic vascular resistance, and enhanced vascular sensitivity to hormonal and sympathetic stimuli [5,6,19,20]. These hemodynamic shifts can modulate the systemic effects of vasoconstrictors, especially if inadvertent intravascular injection occurs during dental anesthesia [5,6,19,20].

Additionally, maternal renal and hepatic clearance are accelerated, potentially shortening the half-life of circulating drugs [20]. Plasma protein concentrations, particularly albumin, are reduced during pregnancy, which may increase the proportion of free, pharmacologically active drug in circulation [20,21,22]. These changes collectively influence the onset, magnitude, and duration of vasoconstrictor action [20,21,22].

The uteroplacental circulation is uniquely susceptible to vasoconstriction. Even transient reductions in uterine blood flow may result in fetal hypoxia, especially in the third trimester when oxygen demands are highest [23,24]. While current evidence suggests that the low systemic absorption of epinephrine used in dental anesthesia rarely reaches clinically significant levels, this margin of safety may be narrower in patients with preexisting cardiovascular instability or obstetric complications such as gestational hypertension or preeclampsia [8,9,10,11,12,13,14,15,16,17,18,19,20,21,22,23,24,25]. In such cases, even mild hemodynamic alterations may translate to disproportionate uteroplacental effects, warranting close interdisciplinary coordination.

On a molecular level, placental transport of vasoconstrictors is modulated by a combination of passive diffusion and active enzymatic degradation. Monoamine oxidase (MAO) and catechol-O-methyltransferase (COMT), present in both maternal and placental tissues, play a key role in metabolizing catecholamines such as epinephrine and levonordefrin [7,26,27]. The efficiency of this enzymatic barrier, however, may vary depending on placental health, gestational age, and maternal disease states such as diabetes or chronic inflammation [7,26,27,28,29,30].

Emerging research in pharmacogenetics further highlights that individual variability in drug response may be influenced by genetic polymorphisms [29,30,31,32,33]. Variants in genes encoding adrenergic receptors (ADRB2 for β2-receptors) and catecholamine-metabolizing enzymes (COMT Val158Met) may alter both systemic reactivity and metabolic clearance of vasoconstrictors [29,30,31,32,33]. While clinical studies in pregnant populations remain limited, these genetic factors could help explain inter-individual differences in hemodynamic response to identical vasoconstrictor doses. In the future, such insights may support personalized anesthetic strategies based on genetic profiling [33].

Taken together, the interplay of pregnancy-induced physiological changes and potential genetic variability underscores the importance of individualized assessment when administering vasoconstrictor-containing local anesthetics. Clinicians must weigh both the pharmacological properties of the agents and the maternal–fetal context to optimize safety and efficacy. These physiological variables make the ultimate effect of vasoconstrictors more difficult to predict in individual patients.

### 4.2. Clinical Risk Stratification and Counseling

The safe administration of vasoconstrictor-containing local anesthetics during pregnancy requires an individualized, risk-stratified approach grounded in interdisciplinary collaboration and informed clinical judgment. Although epinephrine is generally regarded as safe at low doses in dental procedures, not all pregnant patients share the same physiological reserve or risk profile [3,4,7,9,10,11,12,13,14,15]. Effective stratification begins with a comprehensive assessment of maternal health, gestational age, urgency of the dental procedure, and potential contraindications to vasoconstrictor use.

Based on the current understanding of the literature, it is suggested that patients can be broadly categorized into three clinical risk groups. The first group (G1): low-risk patients (LRP), includes those with no comorbidities and uncomplicated pregnancies. These patients tolerate low-dose epinephrine (1:200,000), particularly when administered with aspiration and proper technique. The second group (G2): moderate-risk patients (MRP), includes those with well-controlled chronic conditions (e.g., gestational hypertension, type 2 diabetes, gestational diabetes, other unrelated chronic diseases). These patients may require more vigilant monitoring and, in some cases, pre-procedural obstetric and interdisciplinary consultation. The third group (G3): high-risk patients (HRP), includes those with unstable cardiovascular disease, pre-eclampsia, severe arrhythmias, or recent thromboembolic events. In these cases, vasoconstrictor use should be avoided. Additionally, there is a special group (G4), related to those patients who are allergic to epinephrine (PAE). Also, in this case, the vasoconstrictor should be avoided. We must mention that true allergic reactions to epinephrine are extremely rare; in most cases, hypersensitivity is caused by the metabisulfite antioxidant present in epinephrine-containing solutions. Plain anesthetics such as 3% mepivacaine, which lack preservatives, are safer alternatives in such patients.

**Figure 2:**
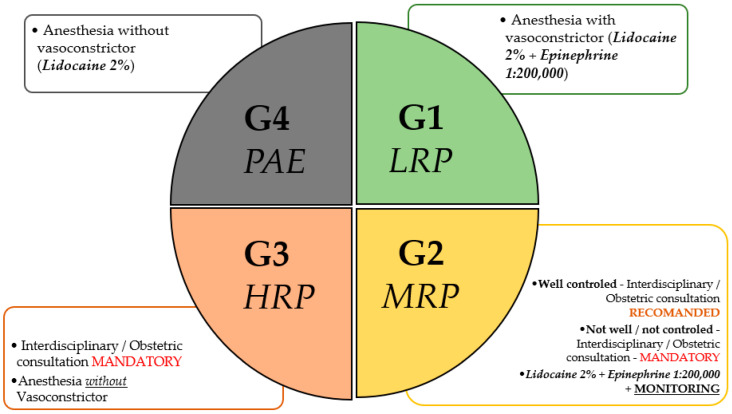
Risk Group Stratification. Risk stratification of pregnant patients for the use of dental anesthesia, based on obstetric assessment. The classification includes the following: G1 (Low-Risk Pregnancy—LRP), G2 (Moderate-Risk Pregnancy—MRP), G3 (High-Risk Pregnancy—HRP), and G4 (Pregnancy After Exacerbation—PAE). G1 allows the use of vasoconstrictors (Lidocaine 2% + Epinephrine 1:200,000 or Articaine 4% + Epinephrine 1:200,000, in the USA) without restrictions. G2 requires monitoring and may permit vasoconstrictors in well-controlled cases. G3 requires mandatory interdisciplinary consultation and restricts vasoconstrictor use. G4 prohibits vasoconstrictors, permitting either plain lidocaine or 3% mepivacaine as a plain anesthetic without additives. Recommendations align with individualized risk–benefit assessments in collaboration with obstetric care providers. The final responsibility for selecting the appropriate local anesthetic lies with the treating dental professional, based on clinical judgment and patient-specific factors.

A summary of the four risk groups regarding the use of vasoconstrictors in dental anesthesia is presented in Figure 2.

The timing of intervention is another key factor. Elective dental procedures are ideally scheduled during the second trimester, when fetal organogenesis is complete and maternal hemodynamic changes are better tolerated [14,15]. However, urgent care should not be delayed if the maternal or fetal risk of untreated oral infection outweighs that of the anesthetic intervention.

To enhance patient safety, procedural precautions must be strictly observed. These include careful aspiration before injection, limiting the epinephrine dose to a maximum recommended dose (MRD) of 0.04 mg per session, using the lowest effective concentration, and employing left lateral tilt positioning in advanced gestation to avoid aortocaval compression [34,35]. Maternal vital signs should be monitored throughout, and post-procedural observation is advisable in moderate- to high-risk cases [36].

Effective patient communication is essential in the dental management of pregnant patients. Counseling should be delivered in clear, empathetic language, addressing both the theoretical risks and the established safety of low-dose epinephrine when administered properly [14,15]. Patients should be informed (verbally or through tailored materials) about the rationale behind anesthetic and vasoconstrictor choices, safety precautions, and the importance of oral health during pregnancy. Informed consent must reflect pregnancy-specific considerations and support shared decision-making [14,15]. In cases involving agents like levonordefrin or felypressin, where human safety data are limited or risks are known, clinicians should clearly explain their avoidance. Ethical practice further requires thorough documentation of the risk–benefit assessment, particularly in medically complex cases [14,15].

**Figure 3:**
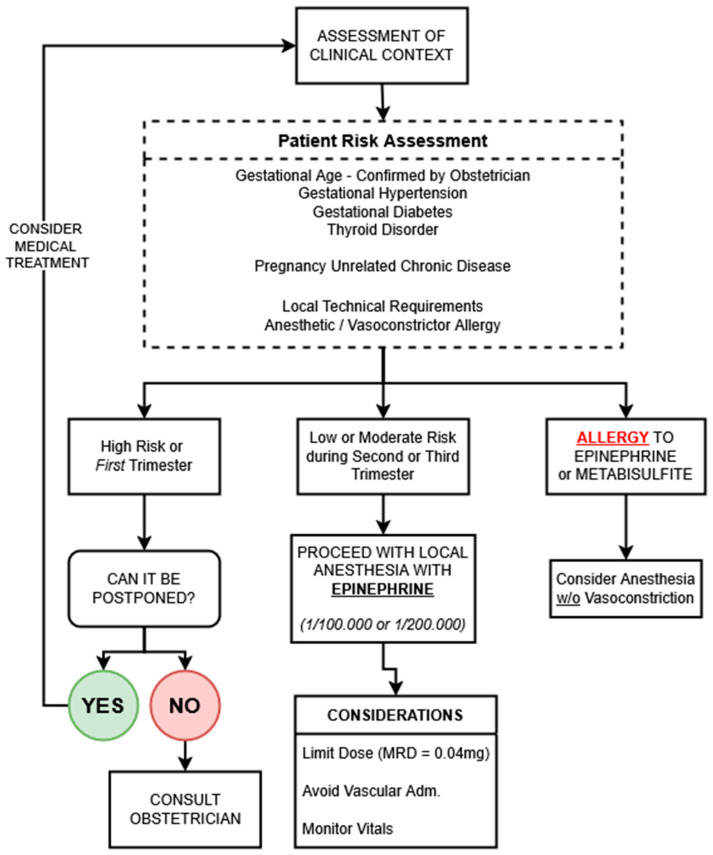
Decision-making algorithm for the use of vasoconstrictor-containing local anesthesia in pregnancy. This pathway integrates clinical context, maternal comorbidities, gestational timing, and anesthetic contraindications to guide safe practice. Low-dose epinephrine is recommended when indicated, with close attention to administration technique and patient monitoring. Obstetric consultation is advised in high-risk or uncertain scenarios. Metabisulfite allergy is rare but may be more common in asthmatic patients. MRD = maximum recommended dose.

A visual summary of the decision-making process is presented in Figure 3 (original figure, comment for reviewer 2), providing a structured algorithm to guide clinical teams in safely navigating dental anesthesia during pregnancy. This tool integrates maternal comorbidities, gestational timing, procedural urgency, and contraindications to support evidence-informed, patient-centered care.

### 4.3. Clinical Evidence: Uterine and Placental Effects of Vasoconstrictors

Well-established clinical studies have demonstrated that epinephrine, when used at concentrations typical for dental anesthesia, does not significantly impair uteroplacental blood flow. For example, the addition of low-dose epinephrine to local anesthetics during labor was not associated with reduced intervillous perfusion in healthy parturients, and in some cases, regional analgesia even improved uterine blood flow [37,38,39]. This paradoxical increase in perfusion may be due to epinephrine’s β2-adrenergic activity, which induces vasodilation in certain vascular beds, including the uterus. These findings are supported by the high metabolic clearance of epinephrine in both maternal and placental tissues, which helps prevent sustained fetal exposure and hypoperfusion [37,38,39].

Although clinical data on vasoconstrictor safety in pregnancy remain limited, preclinical studies provide valuable mechanistic insights into their potential effects on uterine and placental physiology [40,41]. Animal models have demonstrated that epinephrine, at supratherapeutic doses, can induce dose-dependent uterine vasoconstriction, leading to transient reductions in uteroplacental blood flow [40,41]. However, at concentrations equivalent to those used in dental practice, these effects are significantly attenuated. Epinephrine at a 1:100,000 concentration has been shown to be well tolerated when used with proper technique and dose limitation [14,15].

Levonordefrin has been less extensively studied in preclinical settings. Available in vitro and animal data suggest it shares similar α-adrenergic vasoconstrictive properties with epinephrine but may lack the same degree of β-adrenergic modulation [42]. Its effects on the uterine vasculature remain insufficiently characterized, though the theoretical risk of exaggerated vasoconstriction persists in the absence of robust safety data [42].

Felypressin, by contrast, has consistently demonstrated strong uterotonic activity in both in vitro and in vivo studies [12,27,43]. As a vasopressin receptor agonist, it directly stimulates smooth muscle contraction in the uterine wall, significantly increasing the risk of premature labor and fetal compromise, particularly when administered during the second or third trimester [12,27,43]. These findings support the consensus that felypressin is contraindicated in pregnancy.

Epinephrine and levonordefrin differ significantly in adrenergic receptor selectivity, which influences their cardiovascular effects. Epinephrine stimulates both α and β2 receptors equally (~50% each), with β2 activation causing vasodilation that can counteract α-mediated vasoconstriction. In contrast, levonordefrin has stronger α-adrenergic activity (~75%) and limited β2 stimulation, leading to a more pronounced and consistent increase in blood pressure. These pharmacodynamic profiles are clinically relevant in pregnancy, where uteroplacental perfusion is sensitive to vasoconstriction [44,45,46].

While preclinical data cannot replace clinical outcomes, they reinforce the pharmacological rationale for current practice: cautious use of low-dose epinephrine, avoidance of levonordefrin unless absolutely necessary, and strict contraindication of felypressin. Further translational research is warranted to elucidate the dose–response thresholds and placental pharmacodynamics of these agents under varying maternal conditions.

### 4.4. Limitations

The conclusions of this review are subject to several methodological and evidentiary constraints. Most notably, the lack of high-quality prospective clinical trials specifically evaluating vasoconstrictor safety during pregnancy limits the ability to draw definitive, evidence-based recommendations. There are no prospective clinical studies specifically evaluating vasoconstrictor safety during pregnancy; the existing evidence consists primarily of narrative reviews and expert guidelines. In addition, the strong publication bias toward epinephrine studies hinders balanced comparisons, as levonordefrin and felypressin remain poorly represented in clinical data. The exclusive focus on studies written in the English language may have led to the exclusion of relevant studies published in other languages or less prominent journals. Furthermore, the absence of standardized outcome reporting and limited subgroup analysis, particularly for high-risk pregnancies, impairs the ability to fully stratify maternal–fetal risk and limits the generalizability of current findings.

### 4.5. Future Perspectives

To comprehensively address existing knowledge deficits and enhance clinical guidelines, future research initiatives should prioritize several critical areas. Prospective, rigorously designed clinical trials specifically evaluating the pharmacokinetics, placental transfer characteristics, maternal cardiovascular dynamics, and both immediate and long-term fetal outcomes associated with vasoconstrictor use in pregnant populations are urgently needed. Additionally, establishing multicenter registries to systematically collect and analyze clinical outcomes across diverse populations would significantly advance our understanding of real-world safety profiles. Furthermore, investigating emerging alternative vasoconstrictive agents with potentially improved safety profiles, alongside novel non-pharmacological hemostatic techniques such as laser dentistry or topical agents, would greatly expand safe therapeutic options available to clinicians [47,48]. Lastly, developing validated, comprehensive clinical decision-making tools and enhanced interdisciplinary collaborations will be pivotal in optimizing individualized patient care and safeguarding maternal–fetal health.

## 5. Conclusions

Vasoconstrictors remain essential components of dental anesthesia, but pregnancy-specific physiology necessitates a tailored approach. Current evidence supports the cautious use of low-dose epinephrine as the preferred agent, while levonordefrin should be avoided and felypressin is contraindicated.

Future practice should be guided by patient-specific risk profiles, interdisciplinary consultation, and strict adherence to dosing protocols. Strengthening the evidence base, especially for lesser-used agents, remains a key research priority to enhance maternal and fetal safety in dental care.

## Figures and Tables

**Table 1:** Summary of Included Review Articles and Guidelines.

Study/Source and Year	Type of Source	Vasoconstrictor(s) Assessed	Key Findings
Lee and Shin (2017) [7]	Narrative Review	Epinephrine	Low-dose epinephrine (1:100,000–1:200,000) unlikely to cause significant uterine vasoconstriction; rapid metabolism reduces fetal risk.
Lopes and Carneiro (2024) [9]	Review Article	Epinephrine	Supports the use of lidocaine with epinephrine (1:100,000) in pregnancy; highlights relative safety and cautions for other anesthetics.
Fayans et al. (2010) [10]	Review Article	Epinephrine, Levonordefrin	Not recommended in pregnancy due to cardiovascular stimulation and lack of safety data.
Curtin et al. (2022) [11]	Review Article	Epinephrine	Considered safe when administered correctly during pregnancy.
Manautou and Mayberry (2023) [12]	Review Article	Felypressin, Epinephrine	Felypressin not recommended due to oxytocic effects; epinephrine safe at dental doses.
Ho et al. (2021) [13]	Review Article	Epinephrine, Levonordefrin	Summarizes adverse effects of local anesthesia, including systemic reactions and the need for caution in pregnancy.
ADA Clinical Guidelines [14]	Clinical Guideline	Epinephrine	Supports the use of epinephrine-containing anesthetics with proper technique.
ACOG Committee Opinion (2017) [15]	Clinical Guideline	Epinephrine	Dental procedures with epinephrine-containing anesthetics are generally safe if necessary.

This table compiles findings from review articles and clinical guidelines concerning the safety of vasoconstrictor agents (specifically epinephrine, levonordefrin, and felypressin) used in dental anesthesia during pregnancy. Only sources that provided explicit commentary on vasoconstrictor pharmacodynamics, obstetric implications, or fetal risk profiles were included.

**Table 2:** Comparative Pharmacological and Clinical Profile of Dental Vasoconstrictors in Pregnancy.

Parameter	Epinephrine	Levonordefrin	Felypressin
Mechanism of Action	α and β adrenergic agonist	Primarily α adrenergic agonist	Vasopressin receptor agonist (non-adrenergic)
Onset of Action	Rapid (1–2 min)	Rapid (2–3 min)	Rapid (2–4 min)
Duration of Effect	Intermediate (60–120 min)	Intermediate (60–120 min)	Intermediate (60–120 min)
Systemic Absorption	Minimal at dental doses (aspiration reduces systemic exposure)	Moderate (higher systemic absorption potential)	Moderate (similar systemic exposure potential)
Uteroplacental Risk	Low–moderate; dose-dependent vasoconstriction of uterine arteries at higher doses; acceptable at low dental doses	Moderate–high; theoretically more potent uterine vasoconstriction; insufficient human safety data	High; directly stimulates uterine contractions, significant risk of premature labor and fetal compromise
Placental Transfer	Limited placental transfer; rapid metabolism minimizes fetal exposure	Limited data; presumed moderate placental transfer based on structure	Limited data; presumed moderate–high due to smaller molecular size
FDA Pregnancy Category	Category C (risk not ruled out, considered safe at low doses)	Not individually categorized by the FDA; however, 2% mepivacaine + 1:20,000 levonordefrin is Category C in the USA	Not categorized by the FDA; not available in the USA as single or combination product (contraindicated due to known uterotonic risk)
Accessibility	Widely available globally	Less commonly available; limited international usage	Limited availability; primarily in European and Asian markets
Price (Cost)	Low-cost; economical and widely used	Moderate; slightly higher cost, limited market availability	Moderate–high; higher cost due to specialized market
Clinical Guidelines	Endorsed by the ADA and ACOG for use with caution in pregnancy	Not clearly endorsed; guidelines recommend caution or avoidance due to limited data	Contraindicated by clinical guidelines and professional consensus during pregnancy
Cardiovascular Effects	α1 and β2 adrenergic activity; pressor effect moderated by β2-induced vasodilation; well tolerated at low doses	Primarily α1 adrenergic stimulation; limited β2 activity; may cause hypertension with reflex bradycardia	Minimal cardiovascular stimulation; negligible adrenergic activity but risk related to direct stimulation of uterine smooth muscle; risk of preterm labor
Metabolism	Rapid enzymatic degradation (COMT, MAO); very short half-life (~2 min)	Moderate enzymatic degradation (MAO); half-life slightly longer (~2–5 min)	Metabolized rapidly by enzymatic degradation in circulation (vasopressinase); short half-life (~10 min)

Table 2 provides a comparative overview of the pharmacologic and safety profiles of vasoconstrictor agents used in dental anesthesia during pregnancy. Data are synthesized from clinical pharmacology sources, regulatory documents, and peer-reviewed reviews. Where human data were lacking, mechanistic extrapolations based on known receptor activity and metabolism were included.

## Data Availability

The raw data supporting the conclusions and results of this article will be made available upon request.

## Abbreviations

ACOG	American College of Obstetricians and Gynecologists
ADA	American Dental Association
COMT	Catechol-O-methyltransferase
FDA	Food and Drug Administration
ISI	Institute for Scientific Information
MAO	Monoamine Oxidase
MeSH	Medical Subject Headings
MRD	Maximum Recommended Dose
PRISMA	Preferred Reporting Items for Systematic Reviews and Meta-Analyses
